# Application of Ultrasound Pre-Treatment for Enhancing Extraction of Bioactive Compounds from Rice Straw

**DOI:** 10.3390/foods9111657

**Published:** 2020-11-12

**Authors:** Pedro A. V. Freitas, Chelo González-Martínez, Amparo Chiralt

**Affiliations:** Institute of Food Engineering for Development, Universitat Politècnica de València, 46022 Valencia, Spain; cgonza@tal.upv.es (C.G.-M.); dchiralt@tal.upv.es (A.C.)

**Keywords:** bioactive compounds, water extraction, antioxidant activity, pseudo-second order law, antimicrobial activity, heating, combined methods

## Abstract

The extraction of water-soluble bioactive compounds using different green methods is an eco-friendly alternative for valorizing agricultural wastes such as rice straw (RS). In this study, aqueous extracts of RS (particles < 500 µm) were obtained using ultrasound (US), reflux heating (HT), stirring (ST) and a combination of US and ST (USST) or HT (USHT). The extraction kinetics was well fitted to a pseudo-second order model. As regards phenolic compound yield, the US method (342 mg gallic acid (GAE). 100 g^−1^ RS) was more effective than the ST treatment (256 mg GAE·100 g^−1^ RS), reaching an asymptotic value after 30 min of process. When combined with HT (USHT), the US pre-treatment led to the highest extraction of phenolic compounds from RS (486 mg GAE·100 g^−1^ RS) while the extract exhibited the greatest antioxidant activity. Furthermore, the USHT extract reduced the initial counts of *Listeria innocua* by 1.7 logarithmic cycles. Therefore, the thermal aqueous extraction of RS applying the 30 min US pre-treatment, represents a green and efficient approach to obtain bioactive extracts for food applications.

## 1. Introduction

A rising global population and limited natural resources have encouraged the food industries to seek new environmentally-friendly alternatives for agro-industrial waste management [1,2]. One of the primary agro-industrial wastes, rice straw (RS) is a by-product obtained from the rice grain (*Oryza sativa* L.) harvesting, which is considered one of the most important global food crops [3]. Indeed, the Food and Agriculture Organization of the United Nations reported that the annual world rice production was approximately 782 million tons, with 90% production from the Asian continent [4]. Considering that one kilogram of rice grain provides 1.5 kg RS and the largest portion of RS is commonly burnt in the field, there is a continuous increase of air pollution associated with the release of dioxins, affecting human health and reducing the soil feasibility [3,5]. In this way, efforts have recently been made to find new alternatives for the reuse of RS, such as bioethanol production [6], silica extraction [7], paper production [8], cellulose isolation [9] or bioactive compound extraction [10,11].

RS contains about 35% cellulose, 20% lignin, 18% hemicellulose and 15% ashes (dry weight basis) [12]. Likewise, RS has been considered an excellent source of bioactive compounds, including phenolic compounds with antioxidant and antimicrobial potential. Menzel et al. [10] identified bioactive compounds from RS aqueous and alcoholic extracts using ultra-high-performance liquid chromatography coupled to high-resolution mass spectrometry. They found several types of compounds with excellent antioxidant properties, such as ferulic acid, protocatechuic acid, *p*-coumaric acid, caffeic acid, vanillic acid, tricin and vanillin. Some of these phenolic compounds, such as *p*-coumaric, and ferulic acid, have not been found in significant quantities in other vegetable and fruit matrices [13].

In recent years, new technologies have been applied in extraction methodologies in order to overcome problems related to traditional techniques, such as limitations in the extract yield and the fact that they consume a great deal of energy and time [14]. Ultrasound-assisted extraction (UAE) is an eco-friendly and efficient process that applies acoustic cavitation using an ultrasonic probe device in a closed flow-through mode in a determined matrix to extract target compounds. The ultrasound waves, ranging from 20 to 1000 kHz, have been applied to improve the extraction by the solvent acoustic cavitation. The shear forces promoted from the rupture of the bubbles disrupt the cell matrices, extracting higher amounts of compounds by means of different physical phenomena [14,15,16]. Many studies have shown the efficiency of UAE coupled with the greater antioxidant and antimicrobial activity of extracts from different vegetal matrices [17,18,19,20,21]. To the best of our knowledge, UAE has not been previously applied to extract phenolic compounds from the RS matrix to obtain bioactive extracts.

The aim of this study was to improve the water extraction effectiveness of bioactive compounds from rice straw by applying probe-based UAE. The performance of the UAE treatments and their combination with conventional heating and stirring extraction methods was evaluated in terms of the extraction kinetics of phenolic compounds, as well as the antioxidant and antimicrobial activity of the obtained extracts. Likewise, the mass transfer phenomenon was correlated with the possible physical changes that occurred in the ground RS plant matrix.

## 2. Materials and Methods

### 2.1. Chemicals

Gallic acid, Folin-Ciocalteau reagent (2N), methanol, and 2,2-Diphenyl-1-picrylhydrazyl (DPPH) were purchased from Sigma-Aldrich (St. Louis, MO, USA). Sodium carbonate was obtained from PanReac Quimica S.L.U. (Castellar del Vallés, Barcelona, Spain). Tryptone soy broth, tryptone soy agar, phosphate-buffered saline, and peptone water were purchased from Scharlab (Barcelona, Spain). Strains of *L. innocua* (CECT 910) and *Escherichia coli* (CECT 101) were purchased from the Spanish Type Collection (CECT, University of Valencia, Valencia, Spain).

### 2.2. Plant Material Preparation

**Figure 1:**
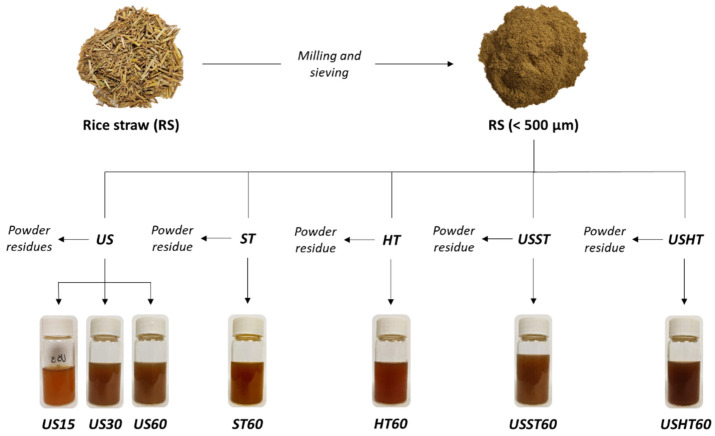
Schematic illustration and appearance of RS extracts obtained from different extraction processes: ultrasound (US), stirring (ST), heating under reflux condition (HT), and a combined treatment (USST and USHT). The number in the sample code indicates the processing time without considering the pre-treatment time.

RS (*Oryza sativa* L.), *J. Sendra var*., was obtained from L’Albufera rice fields, Valencia, Spain, and supply by the “Banco de Paja” (Valencia, Spain), from the harvest 2019. Samples were processed by drying the RS at 50 ± 2 °C under vacuum (0.5 mm bar) for 16 h. After that, the RS was milled using a milling machine (IKA, model M20, Germany) operating in 3 cycles of 90 s each. The ground RS was sieved to obtain particles of under 0.5 mm (representing 98% wt. of the milled RS) (Figure 1) and stored in a desiccator at 20 ± 2 °C until further use.

### 2.3. Aqueous Extraction of RS Powder

The aqueous extracts of phenolic compounds were obtained using an RS: water ratio of 1:20 (m/v). As shown in Figure 1, different extraction approaches were used based on ultrasound (US), stirring (ST), heating under reflux condition (HT), and a combination. The US method was carried out using an ultrasonic homogenizer (Vibra CellTM VCX750, Sonics & Material, Inc., Newtown, CT, USA) equipped with a high-intensity probe operating in a continuous mode. RS and water were mixed and sonicated for 60 min at 25 °C (maintaining the sample in an ice bath to prevent heating), using 750 W power, 20 kHz frequency and 40% sonication amplitude. The ST extract was obtained by stirring the dispersion for 60 min at 350 rpm and 25 °C. The HT method consisted of heating the RS suspension at 100 °C for 60 min using a typical reflux device. Two combined extraction methods were carried out by successively applying 30 min US, as a pre-treatment, and ST for 60 min (USST) or HT for 60 min (USHT). The US pretreatment time was established based on the results obtained for the US treatment. In every process, the supernatant obtained after each treatment was filtered using a qualitative filter (Filter-lab S.A., Cataluña, Spain) and stored in a dark bottle at 4 ± 2 °C until used.

The extraction kinetics was evaluated in terms of the total phenolic content by sampling and analyzing the extract after different process times: 2, 5, 10, 15, 20, 25, 30, 40, 50, and 60 min. To perform the other analyses, extracts obtained from the US method were characterized after 15, 30, and 60 min (US15, US30, and US60), and the ST and HT extracts were analyzed after 60 min extraction time (ST60 and HT60). Likewise, combined USST60 and USHT60 treatments were also characterized. Each treatment was carried out in triplicate and analyzed in terms of total solid yield (TSY), total phenolic content (TPC), antioxidant, and antibacterial activities. TSY was determined by drying the RS extracts at 70 ± 2 °C under vacuum for 6 h until constant weight. After extraction, the residues were collected, and their particle size distribution and microstructure were compared with those of the initial RS powder.

### 2.4. Analysis of Total Phenolic Content

TPC for the different extraction methods over time were determined according to the modified Folin-Ciocalteu method by Menzel et al. [10]. The TPC was determined from the absorbance values, using the linear equation fitted to the stand curve using gallic acid (Abs_725 nm_ = 0.099 (gallic acid) + 0.043; *R*^2^ = 0.9991). The results were expressed as mg gallic acid equivalents (mg GAE) per 100 g of dried RS. All measurements were taken in triplicate.

### 2.5. Modelling Extraction Kinetics

A pseudo-second order rate law was applied to model the extraction kinetics, according to Ho et al. [22]. The dissolution rate of RS compounds, contained within the RS matrix, into water solution can be written as:

(1) $$\frac{d\left(C_{t}\right)}{dt}=k{\left(C_{e}-C_{t}\right)}^{2}$$

where *k* is the second-order extraction rate constant (L·mg^−1^·min^−1^), *C_e_* (mg·L^−1^) and *C_t_* (mg·L^−1^) are the total phenol concentrations in the solvent phase at equilibrium and at any time t (min), respectively. Equation (1) was integrated under the boundary conditions *t* = 0 to *t* and *C_t_* = *C*_0_ to *C_t_*, thus obtaining Equation (2):

(2) $$C_{t}=\frac{ktC_{e}\left(C_{e}-C_{0}\right)+C_{0}}{kt\left(C_{e}-C_{0}\right)+1}$$

when *C*_0_ = 0, Equation (2) can be linearized as Equation (3) and the initial extraction rate, *h* (mg·L^−1^·min^−1^), can be obtained when *t* approaches 0 (Equation (4)):

(3) $$\frac{t}{C_{t}}=\frac{1}{k{\left(C_{e}\right)}^{2}}+\frac{t}{C_{e}}$$

(4) $$h=k{\left(C_{e}\right)}^{2}$$

*C_e_*, *k*, and *h* were determined from the linear fitting of the data in the plot *t*/*C_t_* versus *t*, as inferred from Equations (3) and (4). In the case of treatments applied after 30 min US pre-treatment, when *C_o_* ≠ 0, a non-linear fitting was applied, using the SOLVER tool from EXCEL to obtain *k* and *C_e_* values.

The quality of fit for each extraction method was statistically evaluated by the determination coefficient (*R*^2^) (Equation (4)), and the average absolute relative deviation (*AARD%*) (Equation (5)), which measure the model’s predictive effectiveness.

(5) $$R^{2}=\frac{\sum_{i=1}^{N}{\left(y_{i}^{cal}-y_{i}^{exp}\right)}^{2}}{\sum_{i=1}^{N}{\left(y_{i}^{cal}-y_{m}\right)}^{2}}$$

(6) $$AARD\%=100\times \frac{1}{N}\overset{N}{\underset{i=1}{\sum }}\left|\frac{y_{i}^{cal}-y_{i}^{exp}}{y_{i}^{exp}}\right|$$

where *N* is the number of the data set; $y_{i}^{cal}$ is the predictive value of the model; $y_{i}^{exp}$ is the experimental value; and $y_{m}$ is the mean value.

### 2.6. Antioxidant Activity by DPPH Radical Scavenging Method

The antiradical activities of the different RS extracts were determined using the free radical 2,2-Diphenyl-1-pikryl-hydrazyl (DPPH) method, with some modifications [23]. For each RS extract, different concentrations were mixed with the 6.22 × 10^−2^ mM DPPH methanolic solution (Abs_515nm_ = 0.7 ± 0.2) to a final volume of 4 mL. The initial DPPH concentration in the reaction medium was determined from a calibration curve (Abs_515nm_ = 11.324 [*DPPH*] −0.038; *R*^2^ = 0.9992). The antiradical activity was evaluated by EC_50_, which is defined as the amount of antioxidant necessary to reduce the initial DPPH concentration by 50% when reaction stability has been reached. The reaction stability times between the extract and DPPH solutions were 180 min for the US (15, 30, and 60 min), ST60, and their combined treatment (USST60), and 45 min for the HT60 and USHT60 treatments. To obtain the EC_50_ values, the plots *%* [*DPPH*]*_remaining_* vs. mg solid extract/mg DPPH were obtained, where:

(7) $$\%{\left[DPPH\right]}_{remaining}=\left(\frac{{\left[DPPH\right]}_{t}}{{\left[DPHH\right]}_{t=0}}\right)\times 100$$

[*DPPH*]*_t_* is the DDPH concentration value when the reaction was stable, and [*DPPH*]*_t=0_* is the DPPH initial concentration.

### 2.7. Antibacterial Bioactivity

For both *L. innocua* and *E. coli* strains (stored at −20 °C), a stock solution was obtained by transferring amounts of bacteria strain into 10 mL tryptic soy broth (TSB) using an inoculation loop twice. After incubation at 37 °C for 24 h, the working solution was prepared by pipetting 10 µL of the stock solution in 10 mL TSB and incubating at 37 °C for 24 h. The working solution was serially diluted in TSB to obtain 10^5^ CFU·mL^−1^ (concentration was validated by incubation of 100 µL in tryptic soy agar at 37 °C for 24 h and counting).

The antimicrobial activity of the RS extract against *L. innocua* and *E. coli* strains were analyzed using previously described methods [24,25]. The analysis was carried out in standard 96-well microtiter plates (well volume of 200 µL). For each microorganism, 100 µL of 10^5^ CFU·mL^−1^ bacteria solution was pipetted in each well together with different RS extract solution volumes. Then, the final volume in each well was completed to 200 µL with TSB and incubated at 37 °C for 24 h. The tested concentrations of freeze-dried RS extract dissolved in TSB were 96, 104, 112, 120, 128, 136, 144, 152, and 160 mg/mL^−1^. Typically, the analysis controls were wells containing only bacterial suspension, extract solution and TSB solution. The final counts of each bacteria after incubation were determined in tryptic soy agar media for each well. Thus, 100 µL of each well was transferred in tryptic soy agar plates and incubated at 37 °C for 24 h to obtain the counts. The analysis was performed in duplicate.

### 2.8. Particle Size Distribution in RS Powder

The particle size distributions of RS powders before and after the extraction processes were determined in triplicate using a laser-diffraction particle size analyzer (Malvern Instruments, Malvern, UK) coupled with a Scirocco 2000 dry dispersion unit. A refractive index of 1.520 and an absorption of 0.1 was considered. Samples were fed into the system at a feed rate of 60% and a pressure of 2.5 bar until reaching an obscuration rate of 1.26%. The parameter D_43_ and the mean particle size distribution curves were obtained.

### 2.9. High-Resolution Field Emission Scanning Electron Microscopy (FESEM)

The morphologies of the RS particles after the extraction processes were characterized using a High-Resolution Field Emission Scanning Electron Microscope (GeminiSEM 500, Zeiis, Oxford Instruments, Oxford, UK). Before the microscopy observations, the samples were coated with platinum using an EM MED020 sputter coater (Leica Biosystems, Barcelona, Spain). The images were taken under vacuum and 2.0 kV acceleration voltage.

### 2.10. Statistical Analysis

The data were submitted to analysis of variance (ANOVA), and Tukey’s studentized range HSD (honestly significant difference) test using a Minitab statistical program (version 17). This was performed to determine whether there were significant differences among the extraction methods, using the least significant difference (α) of 5%.

## 3. Results and Discussion

### 3.1. Extraction Kinetics of Phenolic Compounds

**Figure 2:**
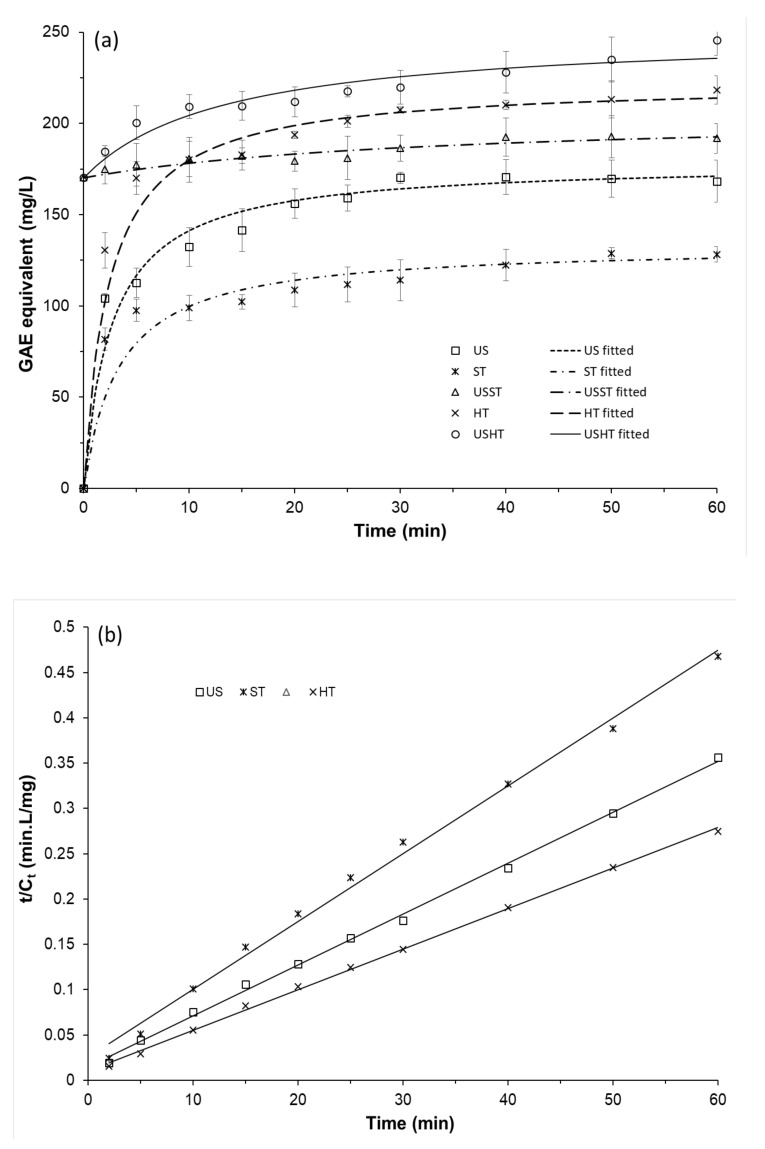
(**a**) Phenolic content of aqueous extracts obtained in the different processes as a function of time (points) and fitted kinetic model (lines). (**b**) Linearization of experimental points from the ST, US, and HT methods according to the pseudo-second order rate law. (US: ultrasound, ST: stirring, HT: heating, USST: stirring with 30 min of US pre-treatment, USHT: heating with 30 min of US pre-treatment).

The performance of the different aqueous extraction methods was compared in terms of the total phenolic content (TPC) reached after different process times (Figure 2a, points). As previously observed by other authors [14,17,26,27] for extraction processes from plant matrices, the compound extraction rate was higher in the first stage of the process (10–15 min), progressively decreasing till the concentration reached an asymptotic value, considered as the equilibrium value (*C_e_*). This is coherent with the greater driving force for mass transfer after short process times [28] while also reflecting the faster initial desorption of compounds from the surface of solid particles and the slower diffusion of compounds extracted from their more internal parts. For the combined USST treatment, the extraction rate was very low throughout the time due to the small difference between the initial TPC concentration (obtained during the 30 min US pretreatment) and that reached at equilibrium, which indicates the limited capacity of simple stirring to improve phenol extraction after the US pretreatment. However, in the USHT treatment, with the same initial TPC value, the equilibrium concentration increased with respect to that obtained in the USST process. Thus, different treatments led to distinct asymptotic values of *C_e_*, depending on the temperature and the application of the US step. Higher temperatures and the US pre-treatment promoted the extraction of the phenolic compounds, giving rise to higher TPC values at equilibrium (*C_e_*).

Therefore, the US method was notably more efficient than the ST treatment (both at 25 °C), the former reaching a higher asymptotic value from practically 30 min extraction time. Thus, 30 min was chosen as the US pretreatment time for combined processes. The combined USST method yielded higher final TPC values than the US, although these values were lower than those reached with a higher extraction temperature (100 °C) in the HT process. The HT extraction behavior suggested that the high temperature significantly favors the release of phenolic compounds from the plant matrix into the extraction solvent, as previously reported by other authors [29,30]. The combination of 30 min US and 60 min HT (USHT process) gave rise to the highest yield in total phenolic content, which indicates the efficiency of the combination of US and thermal treatments as a means of favoring the extraction of phenolic compounds from the RS matrix. Phenolic compounds are linked through acetal, ether or ester covalent bonds with the lignocellulosic fraction of the plant matrix [31,32], and the debonding of phenolic compounds is promoted at high temperatures [33,34]. Thus, the combination of the structural effects provoked by sonication in the solid particles, and the cleavage of phenolic covalent bonds promoted by high temperatures, gave rise to the most efficient method with which to enhance the phenolic extraction yield. Other authors also found an increase in the extraction of phenolic compounds by applying heating or ultrasound to different plant matrices [17,30,33].

### 3.2. Extraction Kinetics Modeling

A pseudo-second order rate equation was adjusted to model the extraction kinetics. The kinetic parameters, the average absolute relative deviation (AARD%), and the determination coefficients (*R*^2^) of the fitted model, are shown in (Table 1). The goodness of model prediction can be seen in Figure 2a, where the experimental points and fitted curves can be observed. Figure 2b shows the fitting of the linearized model when *C_o_* = 0 (cases without US pre-treatment). Likewise, the values of statistical parameters, *R*^2^ (>0.98) and AARD% (<10%), also revealed the goodness of the fitted model, in agreement with other studies [27]. The pseudo-second order rate model is coherent with a two-step mass transfer process for the phenolic compounds: an initial and intensive washing-out of the solute on the RS particle surface, followed by a slow diffusion stage from the interior of the solid particles to the liquid solvent [14,22,28].

For treatments without US pre-treatment (*C_o_* = 0), the initial extraction rates were calculated (*h* values) and are shown in Table 1. The HT treatment presented the highest h value, followed by the US and ST treatments. This finding agrees with the phenol debonding action of high temperatures that promoted phenol extraction from the exposed surface of solid particles. Ultrasonic cavitation at a lower temperature also promoted the fast extraction of phenolic compounds due to the US mechanical action that implied a greater solid surface exposure to the extraction process. However, this effect had a lower impact on the initial extraction rate than the high temperature. The ST treatment exhibited the lowest *h* value, due to the worse ability of low temperatures to debond phenols and the lack of enough mechanical energy to disrupt the particles and increase the extraction surface. The equilibrium concentration values were also positively affected by high temperatures and US application, coherently with their effects on the initial extraction rate (*h*). Thus, the US method increased the phenolic extraction by around 34% with respect to the ST, but it was 24% less extractive than the HT process.

The US pre-treatment (30 min) promoted the phenol extraction in both USST and USHT processes carried out at 25 and 100 °C, respectively. The phenolic equilibrium concentration was increased by around 54% and 13% with respect to that obtained in the ST and HT processes, respectively. Likewise, the *C_e_* value obtained in the USHT treatment increased by around 40% with respect to that obtained in the US treatment, but only by 12% with respect to the HT. This finding agrees with the fact that high temperatures are more effective than ultrasonic cavitation at extracting phenolic compounds. Nevertheless, the combination of both effects in the USHT process produced the maximum extraction of phenolic compounds.

The constant rate values (*k*) were barely affected by either the temperature or the US effect but fell greatly in processes with a US pretreatment (USST and USHT); this was due to the substantial reduction in the process driving force, since the initial concentration of the extract (170 mg·L^−1^) was nearer the equilibrium value.

### 3.3. Changes in the Plant Tissue Produced by Different Extraction Processes

**Figure 3:**
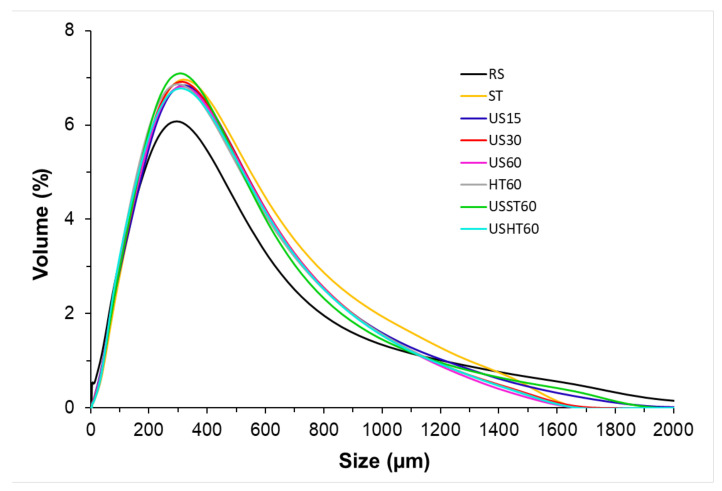
Particle size distribution obtained for the residual powder from the different extraction processes at a determined time (RS: rice straw, US: ultrasound, ST: stirring, HT: heating at reflux, USST, and USHT: combined treatments). The number in the sample code indicates the processing time.

**Figure 4:**
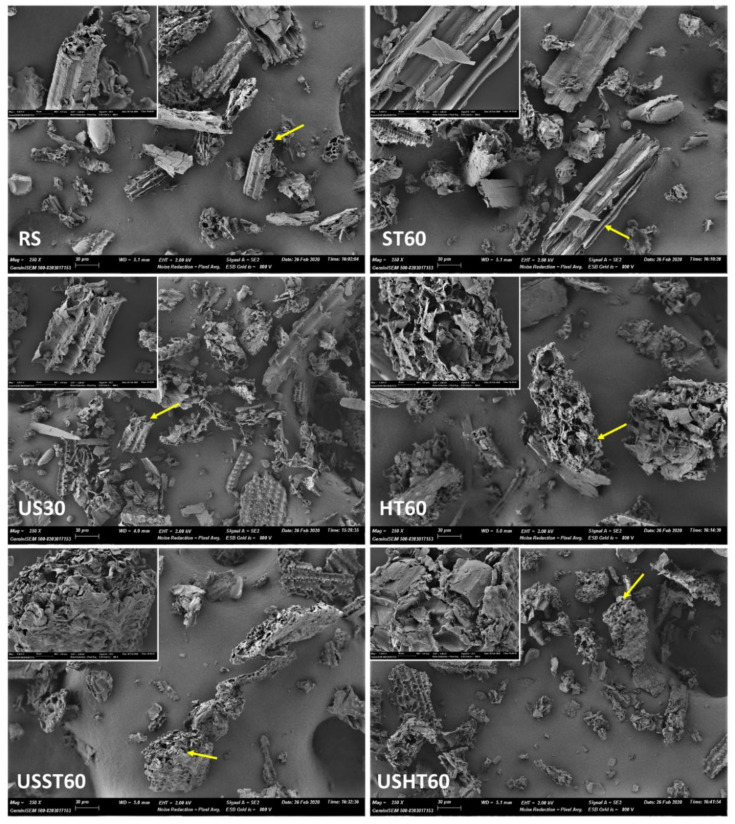
High-resolution field emission scanning electron microscopy (FESEM) images of the particles from the residual powder and untreated RS (1300× magnification). (RS: rice straw, US: ultrasound, ST: stirring, HT: heating at reflux, USST, and USHT: combined treatments). The number in the sample code indicates the processing time. Micrographs of different particles (arrow marked) at higher magnification (×1300) were also included in each case.

Figure 3 shows the particle size distribution of the RS powder and the solid extraction residues after the different treatments: US at 15 (US15), 30 (US30) and 60 (US60) min, ST at 60 min (ST60), HT at 60 min (HT60), and the combined methods (with 30 min US pretreatment) at 60 min (USST60 and USHT60). Before the extraction, the RS particles exhibited a monomodal distribution, with an average particle diameter (D_43_) of 295 µm. As can be observed, the RS distribution showed fractions of the particles with mean diameters larger than the opening size of the used sieve (500 µm). Nevertheless, the RS particles are mostly rod-shaped, with a smaller diameter than length, as shown in Figure 4. Thus, these particles can pass through the mesh even if the length is longer than the opening size. In contrast, the laser diffraction method delivers a particle diameter corresponding to an equivalent sphere with the same diffraction pattern and the length of the particles mainly determines their mean radius of gyration and so, the equivalent sphere diameters.

The extraction processes provoked changes in the particle size distribution to a similar extent in every treatment. The distributions maintained the monomodal characteristic, exhibiting D_43_ values of 308 µm, but were slightly narrower, with a smaller presence of the biggest particles (>1400 µm) and a greater one of particles with an intermediate diameter (300–1000 µm). This behavior was more marked in treatments where the US or high temperatures were applied, suggesting the particles become rounder during extraction. Compared with the other processes, however, it was not especially notable for the US treatments, in which more particle fragmentation would be expected due to the implosion of cavitation bubbles on the particle surface and the inter-particle collisions. Particle fragmentation was found by Machado et al. (2019) in the US extraction of trace metals from globe artichoke leaves and soybean seeds [35]. Nevertheless, US treatments in RS extraction processes could promote other physical changes than particle fragmentation, such as erosion, detexturation, sonocapillarity, or sonoporation, as described by other authors [36,37], which favored the leaching of soluble compounds. Likewise, considering the limitations of the experimental method as a means of characterizing rod-shaped particles, the particle sizes obtained and their potential changes during extraction are not conclusive.

Microstructural changes in RS powders were also analyzed by FESEM (Figure 4) to identify the potential effects of the different treatments on the particles that would explain the different mass transfer patterns. The untreated sample (Figure 4) showed the RS fragments with different dimensions that reveals the original structure of the RS tissue. This has numerous vascular bundles in different layers, consisting of a waxy cuticle, silicate epidermis, cortex (collenchyma), a thick lignified layer (sclerenchyma) and ground tissue (parenchyma) that contained cellulose microfibrils covered with lignin and hemicellulose [38]. Most of the particles resulting from the milling operation exhibit the fibrillar nature of the original tissue. These fibrillar fragments are mainly constituted by the cellulose-hemicellulose-lignin complex where phenolic compounds are bonded. Micrographs obtained at higher magnification were also shown in Figure 4 to better appreciate the morphology of the different particles before and after the extraction process.

From qualitative analyses of FESEM observations (Figure 4), a greater proportion of particles more laminar than tubular in shape appeared in micrographs corresponding to treatments in which US was applied. This suggests a certain degree of plane de-bonding in the fibril structures, resulting from the cavitation phenomena. This fibril destruction would contribute to the promotion of mass transfer during the extraction process in line with the increase of the surface exposed to the solvent action. Other effects, such as sonocapillarity or sonoporation, previously described [36,37], could also occur, but they are difficult to identify in the already unstructured ground tissue. In the thermal treatment without US application (HT60), the presence of flakes or de-bonded lamellas is less noticeable, but particles appeared more distorted, thus revealing the erosive thermal effect associated with the cleavage of phenolic structures from the carbohydrate-lignin complex. Moreover, the more planar particles would have a gyration radius, and thus a mean equivalent sphere diameter, similar to the corresponding original fibrils, which would explain the lack of notable differences in the particle size distribution obtained by laser diffraction in the treatments submitted, or not, to US.

Then, the separation of different planes in the carbohydrate-lignin assemblies provoked by the US and phenol thermal de-bonding as a result of thermal treatment gave rise to the most effective extraction of phenolic compounds when combined in the USHT treatment.

### 3.4. Bioactive Characterization of the Extracts

The RS extracts obtained from different extraction methods were characterized in terms of total solid yield (TSY), total phenolic content (TPC), antioxidant (Table 2), and antibacterial activities. RS contains a wide variety of compounds, such as lignin, cellulose, tannins, hemicellulose, sterols, flavonoids, phenolic aldehydes, and hydroxycinnamic acids, which may or not have bioactive properties [11,12,21]. The amount and proportion of these extracted compounds depend strictly on the extraction parameters, such as the method, type and proportion of extractor solvent, time, and temperature.

As shown in (Table 2), non-statistical differences were found between the TSY values of ST60 and US15 treatments (*p* > 0.05) while the total solid extraction efficiency of the US treatment significantly increased (*p* < 0.05) when using longer sonication times, as has been previously observed by other authors [29]. No statistical differences were observed (*p* > 0.05) between the TSY of the US60 and HT60 treatments carried out at 25 and 100 °C, respectively. However, although the effects induced by ultrasonic cavitation and heating were comparable in terms of the total water solubilization capacity of the compounds present in the RS matrix, differences in the extract composition could provide distinct bioactive properties. The combination of US and heating (USHT60) yielded the highest solid leaching from RS (*p* < 0.05). Thus, as previously commented on, the US pre-treatment led to there being more points of the RS structure accessible to the solvent, thereby improving the mass transfer potential of RS compounds. The US pre-treatment increased the total solid leaching by around 60% and 45% when used before the stirring and heating processes, respectively. Heat treatment provokes the softening of the plant tissue, weakening the cell wall integrity and the bond hydrolysis of the phenolic compounds linked to the carbohydrate-lignin complex, thus enhancing the solubility of phenols and other compounds [29,36,37].

The total phenolic content of the different extracts was expressed with respect to the RS mass (TPC_1_) and to the total extracted solids (TPC_2_). The former permits the observation of what treatment was more effective at extracting phenolic compounds from the raw material, whereas the latter supplies information on how rich in phenolic compounds the different extracts, which also contain other soluble compounds, are. Thermal treatments at 100 °C, with and without US pre-treatment, yielded the highest extraction of phenolic compounds from RS, according to what is described by other authors when analyzing the temperature effect on the phenolic de-bonding from the cellular structure. The US pre-treatment enhanced this effect in line with the increase in the solid matrix surface available for the extraction, provoked by the cavitation phenomenon. The US pre-treatment improved the TSY of the ST treatment and the extraction of phenolic compounds from the RS matrix, but the extract’s richness in phenols was slightly reduced due to the simultaneous promotion of the extraction of other components. In the same way, in the HT process, although the US pre-treatment enhanced the phenolic extraction, it led to the release of a greater quantity of other solids, thus implying an increase of 45% in the total solid extraction, but of only 6% in the phenolic compounds from the RS. The TPC values of the extracts (TPC_2_) revealed that the greater total solid extraction does not guarantee a higher content of phenolic or active compounds in the extract. Thus, similar TPC_2_ values were obtained for the US60 and HT60 treatments despite the different total solid yield. The poorest extract in phenols (lowest TPC_2_ value) was that of the USHT60 treatment with the highest TSY. Therefore, what this treatment promoted was the extraction of non-phenolic compounds as opposed to phenolic. The extracts from the US15 and US30 treatments had similar phenolic contents (TPC_2_ values), however, longer US treatment times slightly decreased the phenolic richness of the extracts, thus indicating a greater promotion of the extraction of non-phenolic compounds. In this sense, other authors [39] indicated that 20 min of sonication time was enough to extract phenolic compounds from soy beverages.

A DPPH radical scavenging assay was used to analyze the antioxidant activities of the RS extracts obtained from different extraction methods. The results were expressed in terms of the EC_50_ parameter, referred to the mass of RS and extract (EC_50(1)_ and EC_50(2)_, respectively). Thus, EC_50(2)_ indicates the radical scavenging capacity of the extracts, whereas EC_50(1)_ reflects the ability of the extraction method to extract compounds with more radical scavenging capacity from the RS. A significant correlation was observed between the capacity of the extraction method to obtain phenolic compounds from RS (TPC_1_) and the radical scavenging capacity (EC_50(1)_), with r values of −0.960. This indicates that the treatments that extracted a greater quantity of phenolic compounds from RS also extracted more compounds with radical scavenging capacity, which can be mainly attributed to the phenols. However, the parallel extraction of other compounds, as affected by the kind of treatment, determined the final ratio of the phenols in the extracts and their EC_50(2)_ values. As can be observed in Table 2, the ST60 exhibited the lowest radical scavenging capacity (the highest EC_50_ values). Other authors [10] found a similar EC_50(2)_ value (12.0 mg freeze-dried extract/mg DPPH) when extracting compounds from RS under similar conditions. Although the US treatments promoted the enrichment of the extracts in non-phenolic compounds when the extraction time lengthened, the extracts for 15, 30, and 60 min exhibited a similar radical scavenging capacity (*p* > 0.05), which was greater than that of the ST extract. The combination of the US with ST (USST60) did not imply extracts with more radical scavenging activity than that obtained by the US pre-treatment, nor was the extract enriched in phenolic compounds. In contrast, the thermal treatment produced the extracts with some of the highest radical scavenging capacities; this was enhanced by the US pre-treatment, despite the fact that US application more effectively promoted the extraction of non-phenolic compounds and, globally, the USHT60 extract had the lowest total phenolic content. This finding indicates that the thermal treatment promotes the extraction of the more active compounds (phenolic or not); this was enhanced when US pretreatment was applied in line with the higher surface exposure in the solid particles due to the cavitation phenomenon. Other authors [30] also observed that the microwave and conventional oven heating pre-treatments promoted a significant increase in the antioxidant activity of extracts from fennel seeds, which was attributed to the promotion of flavonoid extraction. Flavonoid extraction from baobab seeds was also promoted when US treatments were carried out at higher temperatures [17]. Several compounds with proven antioxidant capacity, such as ferulic, protocatechuic, *p*-coumaric, caffeic, and vanillic acids, tricin, and vanillin have been identified in RS extracts by other authors [10,40].

In brief, the obtained results suggested that the combined ultrasound-heating method promoted a greater extraction of compounds from RS with antioxidant properties, diluted with other non-phenolic components, and, from a practical point of view, the obtained USHT60 extract exhibited the best potential to act as an efficient antioxidant material.

Considering the ability of the US and HT combination to extract bioactive compounds in terms of the radical scavenging capacity, the corresponding extract (USHT60) was also evaluated as to its antibacterial activity against *L. innocua* and *E. coli* strains. The extract obtained in the corresponding US30 pretreatment (richer in phenolic compounds) was also analyzed. Thus, different concentrations of freeze-dried extracts (96–160 mg·mL^−1^ TSB) were applied in the well microtiter plates in order to determine the minimum inhibitory concentration. No total inhibition of bacterial growth was observed for the extract over the considered concentration range. However, the most heavily-concentrated USHT60 extract at 160 mg·mL^−1^ reduced the initial counts of *L. innocua* by 1.7 log CFU, although it was not effective at reducing the counts of *E. coli*. At the same concentration, the US30 extract was not effective at reducing the initial counts of *E. coli* or *L. innocua*, which suggests that the extraction of more active compounds was achieved during the thermal step of the extraction process. Although extracts obtained from RS contain antimicrobial compounds, such as ferulic and *p*-coumaric acids [10,21] with proven antibacterial activity against these strains, their concentration could not reach the minimal inhibitory concentration of the bacteria, or their interactions with other extract components could limit their effectiveness as antibacterial agents.

## 4. Conclusions

RS is an agro-industrial waste that contains a wide variety of bioactive compounds of technological interest for food application purposes, but the extraction conditions greatly influence the extraction efficiency of the target compounds. The application of ultrasound was notably more effective at extracting water-soluble phenolic compounds than simple stirring, as revealed by the higher extract yields and antioxidant activity of the extracts. Nevertheless, a high extraction temperature produced materials with improved antioxidant activities, in line with the promotion of the cleavage of covalent bonds between the phenolic compounds and the lignocellulosic fraction. This thermal effect was greatly enhanced when ultrasound pretreatment was applied due to the increase in the substrate surface exposed to the extraction. Therefore, a combination of 30 min US plus 60 min thermal treatment in water reflux is recommended to obtain solid extracts with great antioxidant activity and notable anti-listerial effect, which could be used in the food or pharmaceutical industries.

## Figures and Tables

**Table 1:** Kinetic parameters obtained for the different extraction processes.

Extraction Method ^1^	*k* (× 10^−3^) (L·mg^−1^·min^−1^)	*C_e_* (mg·L^−1^)	*h* (mg·L^−1^·min^−1^)	*R* ^2^	*AARD*%
US	2.12	178.6	67.60	0.998	4.89
ST	2.24	133.3	39.82	0.994	7.76
HT	1.91	222.2	94.32	0.992	4.08
USST	0.84	205.3	-	0.980	1.14
USHT	0.93	250.3	-	0.985	2.14

^1^ ST: stirring, US: ultrasound, HT: heating, USST and UHHT: combination of 30 min US and ST or HT, respectively.

**Table 2:** Total solid yield (TSY), total phenolic content (TPC) and antioxidant capacity (EC_50_) of the different extracts. Phenolic content and antioxidant capacity were expressed per mass unit of RS and dry extract.

Method ^1^	TSY (g Dry Extract. 100 g^−1^ RS) *	TPC_1_ (mg GAE 100^−1^ g RS) *	TPC_2_ (mg GAE. g^−1^ Dry Extract) *	EC_50(1)_ (g RS. mg^−1^ DPPH) *	EC_50(2)_ (mg Dry Extract. mg^−1^ DPPH) *
ST60	5.61 ± 0.07 ^a^	256 ± 3 ^a^	45.7 ± 0.1 ^a,b^	19.7 ± 0.6 ^a^	11.0 ± 0.5 ^a^
US15	5.7 ± 0.3 ^a^	284 ± 17 ^b^	49.2 ± 0.9 ^a^	16.0 ± 1.3 ^b^	9.2 ± 0.3 ^b^
US30	7.48 ± 0.05 ^b^	342 ± 10 ^c^	45.7 ± 1.0 ^a,b^	12.5 ± 0.1 ^c^	9.35 ± 0.18 ^b^
US60	9.48 ± 0.17 ^c^	354 ± 12 ^c^	37.4 ± 1.9 ^c,d^	9.8 ± 0.3 ^d^	9.3 ± 0.3 ^b^
HT60	9.6 ± 0.6 ^c^	459 ± 6 ^d^	47.0 ± 3.0 ^a^	7.8 ± 0.5 ^e^	7.49 ± 0.05 ^c^
USST60	9.0 ± 0.4 ^c^	382 ± 6 ^e^	42.3 ± 2.4 ^b,c^	10.4 ± 0.3 ^f^	9.39 ± 0.10 ^b^
USHT60	13.95 ± 0.13 ^d^	486 ± 4 ^f^	34.8 ± 0.5 ^d^	4.6 ± 0.3 ^g^	6.3 ± 0.4 ^d^

^1^ ST: stirring treatment, US: ultrasound treatment, HT: heating treatment, USST and UHHT: combination of 30 min US, and ST or HT, respectively. The number in the sample code indicates the process time. * Different superscript letters in the same column indicate significant differences (α = 0.05).
